# Green Initiatives and Environmental Concern Foster Environmental Sustainability: A Study Based on the Use of Reusable Drink Cups

**DOI:** 10.3390/ijerph19159259

**Published:** 2022-07-28

**Authors:** Xiuting Wang, Idrees Waris, Muhammad Yaseen Bhutto, Haowei Sun, Irfan Hameed

**Affiliations:** 1 School of Management, Wuhan University of Technology, Wuhan 430070, China; wxt660@126.com; 2 Department of Management Sciences, University of Turbat, Turbat 92600, Pakistan; idrees.waris@uot.edu.pk; 3 Business School, Shandong Jianzhu University, Jinan 250101, China; 4 School of Art, Shandong Jianzhu University, Jinan 250101, China; sunhaowei@sdjzu.edu.cn; 5 College of Business Management, Institute of Business Management, Karachi 75190, Pakistan; irfan.hameed@iobm.edu.pk

**Keywords:** green university initiatives, environmental concern, moral norms, perceived behavioral control, attitude, subjective norm, reuse intention

## Abstract

Unsustainable production and consumption have threatened human life and nature. Therefore, practitioners around the globe have paid attention to sustainability issues and adopted pro-environmental strategies to protect the environment. Using single-use cups contributes to environmental pollution. This study aims to understand university students’ intention to use reusable drink cups in university campuses. This study has extended the theory of planned behavior (TPB) model by including moral norms, green university initiatives, environmental concerns, and moral norms. The purposive sampling technique has been employed to collect students’ data from the twelve universities in Pakistan. Partial least square structural equation modeling (PLS-SEM) has been employed to test the hypothesized model. The study’s results revealed that green university initiatives and norms significantly shape students’ intention to use reusable cups. However, environmental concern has an insignificant impact on the perceived behavioral control. This study’s results help higher education institutions to formulate strategies that create awareness among students and promote environmentally sustainable practices.

## 1. Introduction

The fast-growing population has contributed to world pollution and forced policymakers to consider sustainable waste management [1]. The excessive use of paper cups contributes to environmental pollution [2,3]. It is estimated that nearly 16 billion paper cups are discarded yearly [4]. Another study indicated that end-users generated 20 times more plastic than 5 million tons in the 1950s [1]. The overconsumption and excessive use of plastic surpassed 297.5 million tons in 2016, and it is expected to increase by 12 billion tons by 2025 [5]. Around 11% of plastic waste that is produced worldwide is moved directly into the water ecosystem [6]. Researchers highlighted that using a single cup for takeaway beverages is one of the main problems of plastic pollution due to a thin layer of plastic inside the cups that makes the single-use cup unsuitable for recycling [7,8]. Plastic does not absorb water as it is made of synthetic organic materials that are produced by polymerization that pollute the environment [9]. Therefore, reusable drink cups are a viable option to promote environmental sustainability as they are made of glass, metal, and durable plastics [10].

Policymakers in advanced countries are more concerned about managing plastic waste than in developing nations [5]. The rapid industrialization in developing countries has also contributed to solid waste production with serious ecological consequences [11]. Poor infrastructure and the unavailability of resources have resulted in improper waste management. Wastes’ mismanagement and its dumping in the slums have caused severe health and environmental issues in developing countries. Municipalities in developed countries provide door-to-door waste collection facilities, but this facility is limited to a few areas in developing countries due to financial constraints [12]. As a result, the wastes are thrown into slums and dumpsites that cause serious health and environmental threats to the people [13]. Past studies have examined the impact of waste mismanagement and concluded that waste and environmental health are closely interlinked [11]. Over the years, researchers focused on the end-users regarding the recycling of waste products in developed and developing countries such as England [14], India [15], and Pakistan [5]. In addition, some recent studies have focused on people’s intention to use reusable drink cups and recycle plate waste to promote environmental health [3,10]. Pakistan, one of the developing countries, is also far behind in managing waste products [11,16]. Due to non-effective recycling systems in Pakistan, pollution has been getting worse over the years [17].

Further, when paper wastes are set on fire or dumped on the land they get chemically toxic [5]. The government of Pakistan enacted the climate change act in 2017 to deal with environmental pollution. However, Yale’s Environmental Performance Index (2018) has ranked the country 169 out of 180 [17]. Government policies alone do not produce the desired results unless there is a concentrated effort from the end-users [5]. In this regard, universities have a pivotal role in educating and imparting a sense of ownership among the students to promote environmental health. Researchers indicated that Green university initiatives (GUIs) aim to create awareness and encourage sustainability on campus [18]. 

GUIs include awareness programs to educate the students on sustainable developments that are aimed at reducing the adverse effect of unsustainable consumption while raising students’ moral obligations toward sustainability. For example, students’ use of technology will reduce the negative impact on the environment, thus helping shape pro-environmental behavior [19]. In addition, the University premises offer opportunities for fostering sustainable learning [20]. Researchers indicated that students need to be aware of three points: first, renewable resources play an essential role in sustainability; second, sustainable development is associated with social development; and third, a part of income should be allocated for the betterment of society [21]. Other researchers suggested that campus activities such as lectures, research, sustainable development workshops, and outreach could influence students’ behavior. Further, it is also essential that universities should advantage of sustainable initiatives [22,23] and engage the students in sustainability-related activities [24]. Researchers highlighted the vital role of the university in developing sustainable activities and imparting a sense of responsibility among the students to protect the environment [21,23]. Higher education institutions practicing green initiatives can accomplish environmental and social goals [18,21]. Researchers suggested that universities focus on pilot projects with awareness campaigns to highlight the environmental issues that lead to attaining green campus goals [22]. 

Past studies have focused on systematic interventions to promote reusable drink cups [3,10,25]. Many studies have been conducted to understand reusable drink cups or bottles [2,3]. Mainly, the impact of GUIs on students’ pro-environmental behavior in the context of a developing country has been ignored in past studies. Therefore, this study fills the gaps by understanding university students’ intentions to use reusable drink cups through the lens of an extended theory of planned behavior (TPB). Understanding the role of GUIs, environmental concerns, and moral norms in shaping students’ intentions is essential to promoting the use of reusable drink cups that would positively impact the environment. 

This study extends the theory of planned behavior by adding GUIs, environmental concerns, and moral norms. Researchers highlighted the critical role of moral norms in the TPB model and argued that including moral norms increases the variance in behavioral intention [26,27]. Notably, some researchers argued that adding moral norms is vital in the case of pro-environmental behavior [28,29]. Therefore, understanding the impact of moral norms on students’ reuse of drink cups is essential to promoting environmental health. Very few studies have assessed the impact of UGIs’ on sustainable behavior [21,22,30], but UGIs are rarely evaluated in the context of reusable products. Hence, UGIs are a novel addition contributing to the extension of the theory of planned behavior. This study would be helpful for the managers and practitioners as it would contribute to the comprehensive understanding of university students’ intention to reuse drinking cups on university campuses. 

### 1.1. Literature Review

#### Theoretical Background: Theory of Planned Behavior (TPB)

**Figure 1:**
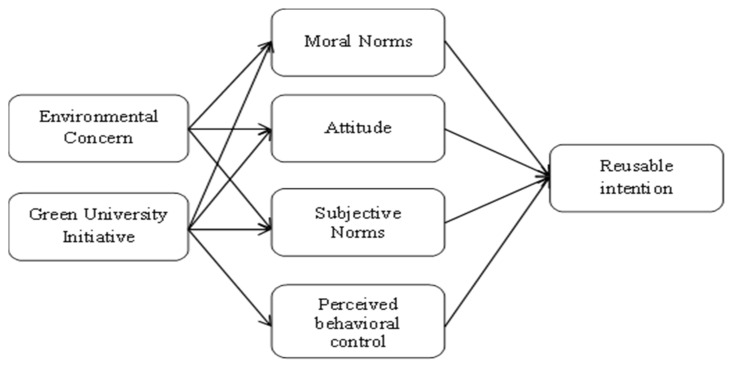
Conceptual Model.

The theory of planned behavior (TPB) has been used to better understand the influence of green university initiatives and environmental concern on the TPB constructs. Ajzen’s [31] TPB helps to systematically evaluate the predictors that influence intention, including sustainable behavioral intention [32,33]. Therefore, this study employed TPB to evaluate university students’ intention to use reusable drink cups. The TPB is an extension of the theory of reasoned action (TRA), which is widely used to predict behavioral intention in sociology and psychology [34]. The theory of planned behavior (TPB) includes three distinct constructs that assess people’s behavioral intentions: attitude, perceived behavioral control, and subjective norms. In many studies, the importance of the theory of planned behavior has been successfully applied and validated by researchers [35,36]. Researchers have extended TPB and successfully evaluated behavioral intentions in various studies, such as assessing people’s intention to use reusable products [5,36], resident’s vehicular PM2. 5 reduction intention [37], hybrid electric car purchase intention [29], willingness to visit green hotels [38], buying organic foods [39], and energy-efficient appliances purchase intention [40]. The additional aspects in the theory of planned behavior have enhanced human behavior in their respective fields. Ajzen [31] also suggested that TPB is vital in evaluating people’s behavioral intentions. Further, he suggested adding TPB to better assess the behavior by considering the following points: (1) the additional building should serve the aim of logical decision-making. (2) In the new model, new constructs should be free. (3) A particular behavior should be predicted by the additional constructs. Therefore, this study has extended TPB by adding the GUIs, environmental concerns, and moral norms in TPB to evaluate university students’ intention to use reusable drink cups. The conceptual framework is shown in Figure 1.

### 1.2. Attitude (ATD)

Attitude refers to a positive or negative evaluation of a person, object, and situation [31]. The theories of reasoned action and planned behavior posit that individual behavior results from a favorable attitude [41]. From the perspective of pro-environmental behavior, researchers argued that a positive attitude leads to behavioral intention [42,43,44]. For instance, several studies have proven that a positive attitude is an essential antecedent of behavioral intention, such as in the recycling and reuse of plastic wastes [5], e-waste disposal behavior [45], recycling of solid waste [46], the purchase of energy-efficient appliances [41], mobile waste recycling [42], and suboptimal foods [43]. Specifically, studies revealed that attitude has a positive influence on the intention to reuse drink cups and straws [3,10,25]. Based on previous studies, we can assume that attitude is an essential antecedent of university students’ intention to use reusable drink cups. Hence, we propose that:

**Hypothesis** **1** **(H1).** *Attitude will positively influence students’ intention to use reusable drink cups.*

### 1.3. Subjective Norms (SN)

A subjective norm refers to the influence of society and social circle in performing a particular behavior [5]. In other words, it refers to the perceived social pressure that an individual opts to achieve a particular act [31]. In the context of environmental studies, researchers found that the subjective norm is an influential factor that affects behavior. For example, researchers found that the influence of peers and friends affect recycling intention [5], purchase of energy-efficient appliances [41], and electronic-waste recycling [45]. A recent study that was conducted in China and Finland on waste sorting and recycling found a significant impact of subjective norms on behavioral intention [46]. From the developing nations perspective, researchers used an extended TPB model and found the significant impact of subjective norms on recycling plastic waste [5,47]. Recently, researchers found that subjective norms positively influence individual intention to use reusable coffee cups [3,7]. Past studies’ findings provide enough support regarding the positive influence of subjective norms on intention. Therefore, we can assume that subjective norms positively influence the recycling of plastic waste. Hence, we propose the following hypothesis:

**Hypothesis** **2** **(H2).** *Subjective norms will positively influence students’ intention to use reusable drink cups.*

### 1.4. Perceived Behavioral Control (PBC)

Perceived behavioral control refers to the individual’s belief that he/she can easily perform a particular behavior. PBC is the perceived ease or difficulty of performing a task based on previous individual experience and anticipated hurdles [31]. It assesses the individual’s ability to overcome obstacles while performing the task [48]. In the environmental domain, people better deal with constraints and difficulties and exhibit higher behavioral intentions [49]. The extant literature shows that perceived behavioral control is an essential driver of people’s behavioral intention [34,41,45]. In the context of environmental studies, researchers argued that it is an essential element that drives the purchase intention of hybrid vehicles [29,50], purchase of skincare products [51], shared electric bicycle intention [52], green product purchase [26], and energy-saving behavior [53]. Many researchers have found the individual’s behavioral control to be an essential determinant of behavioral intention [27,29,31]. Past studies have demonstrated that perceived behavioral control is a crucial element of the TPB model that significantly affects the use of reusable bottles [54], reusable drinking straw [25], and anti-PM2.5 mask and filter behavioral intentions [55]. Based on the previous studies’ results, we can assume that perceived behavioral control will positively influence university students’ intention to use reusable cups on university campuses. Therefore, we propose the following hypothesis:

**Hypothesis** **3** **(H3).** *Perceived behavioral control will positively influence students’ intention to use reusable drink cups.*

### 1.5. Moral Norm (MN)

Moral norm is a psycho-social factor that has been given ample attention by environmentalists [56]. It is an intrinsic quality of a person depicted through moral standards and judgments towards certain activities [57]. People who develop moral norms have a deep concern for environmental sustainability [58]. The research highlighted the importance of moral norms as a motivational construct in the TPB model [59,60]. Toward going for a particular behavior, if the individual thinks that the outcomes of doing a particular behavior are positive, then that person shows a positive attitude [5]. Ajzen’s (1991) TPB model has been criticized due to the absence of moral obligation by the individuals [31]. Therefore, moral norms or obligations are considered essential to pro-environmental behavior [59,60]. In line with this, several authors also argued the importance of moral norms and posited that moral norms are essential determinants of behavioral intentions [61,62]. Based on the above assumptions, we can assume that moral norms significantly affect students’ intention to use reusable drink cups. Hence, we propose the following hypothesis:

**Hypothesis** **4** **(H4).** *Moral norms will positively influence students’ intention to use reusable cups.*

### 1.6. Environmental Concern (EC)

Environmental concern refers to people’s awareness regarding the environmental issues [63], and their efforts and inclination to contribute to the solutions [34]. Researchers defined a broader definition of environmental concern that includes specific environmental attitudes and value orientation [64]. Environmental concern is “an affective attitude regarding the severity of environmental problems” [63]. Studies have revealed that people with environmental concern are committed to solving environmental issues and would exert influence on others to do same [64,65]. Researchers used an extended TPB model and found that environmental concern exerts a positive influence on ATD, SN, and moral obligation in the intention to visit green hotels [38]. A study revealed that environment concern has a strong positive influence on ATD and SN, regarding the acceptance of bicycle-sharing [65]. Past studies have confirmed the positive impact of environmental concern on ATD, SN, and moral norms [26,29,64]. Based on the past studies evidence regarding the efficacy of environmental predicting pro-environmental intention, we can assume that environmental concern exerts a positive influence on the extended TPB constructs. Hence, we propose the following hypotheses:

**Hypothesis** **5** **(H5).** *Environmental concern will positively influence attitude.*

**Hypothesis** **6** **(H6).** *Environmental concern will positively influence subjective norm.*

**Hypothesis** **7** **(H7).** *Environmental concern will positively influence moral norm.*

### 1.7. Green University Initiatives (GUIs)

GUIs are a holistic concept that encapsulates environmental awareness and practices at higher education institutions [66]. In the last two decades researchers have used different terminologies such as “sustainable university”, “green university”, and “Green campus” to assess the efforts of universities in promoting sustainable development [18]. To promote sustainable development, researchers designed a balanced scorecard strategy map that help to establish and trace the impact of educational programs among the campus users [67]. HEIs have a vital role in implementing and promoting sustainable practices, designing curricula, and research and development programs [68]. Green initiatives are the programs that are initiated to foster pro-environmental practices [69]. HEIs sustainable development goals are gradually increasing and leading to the development of sustainable societies [70,71]. The extant literature reveals that higher education institutions have implemented environmental policies and daily activities in the university premises [72,73]. Some green practices that universities have adopted include energy conservation, reducing carbon footprint and greenhouse gas emission, water conservation activities, and adopting green purchasing policies [74,75]. GUIs impart awareness through the promotion of efficient utilization of resources such as efficient energy management practices [76]. Researchers highlighted that university’s green initiatives and environmental concern are essential factors that lead to pro-environmental behavior [68,77,78]. Researchers encouraged the users to practice sustainable development in campus by saving energy and water, using sustainable transportation, separating wastes, and promoting social development [18]. Based on the above evidence, we can assume that GUIs are essential predictors of the extended TPB model in predicting students’ intention to use reusable cups. Hence, we propose the following hypotheses:

**Hypothesis** **8** **(H8).** *A Green university initiative will positively influence attitude.*

**Hypothesis** **9** **(H9).** *A Green university initiative will positively influence the subjective norm.*

**Hypothesis** **10** **(H10).** *A Green university initiative will positively influence the perceived behavioral control.*

**Hypothesis** **11** **(H11).** *A Green university initiative will positively influence the moral norm.*

## 2. Materials and Methods

### Sampling

In this study, a self-administered questionnaire was used as the instrument for data collection. The respondents have been selected from the five main cities of Pakistan (Karachi, Lahore, Islamabad, Hyderabad, and Peshawar). The purposive sampling technique has been employed for the collection of data. The first section of the questionnaire was related to the respondents’ demographic information. The second section was related to the items of the constructs. The primary purpose of the research was mentioned at the start of the questionnaire, and the participants were requested not to mention their identities. The sample size for this research was determined by following the guidelines of researchers [79]. They suggested selecting ten responses per item. As the total number of items was 32, we required 320 samples. To increase the reliability and make the data normal, we have distributed the questionnaires to 550 respondents. Data collection was carried out from 21 January 2022 to 15 May 2022. The students were approached in the classes and canteens of twelve universities in Pakistan. Many students were reluctant to fill out the questionnaire during the survey due to their busy schedules. In addition, we received many unfilled and incomplete questionnaires that were discarded before analysis. After discarding the incomplete questionnaires, 361 valid responses were taken for final analysis. The response rate was 65.63%. Before the final analysis of the data, we performed a multivariate analysis test to exclude the outliers from the data; there were three outliers that were detected and eliminated from the dataset. The details of the respondents’ profiles are mentioned in Table 1. All the constructs’ items are adopted from previous studies. The details of the measurement scale and their sources are mentioned in Table 2. 

## 3. Results and Analysis

The results of the current study were analyzed through SPSS and SmartPLS. SPSS has been used to identify multivariate outliers and assess the common method bias. To confirm the existing theory, the PLS-SEM is the most suitable tool [84]. It took two stages. At first, the model fitness indicators were identified with reliability and validity. In the second stage, the proposed hypotheses’ results were obtained by applying the 2000 bootstrapping method.

### 3.1. Common Method Bias

The purification of data was performed to avoid the common method bias. Common method bias is a threat to the credibility of the collected data. Therefore, it suggested ensuring that the data are free from common method bias [85]. For this purpose, we have used Harman’s single factor test. If the total variance that is explained by one factor is less than 50%, then data are free from the threat of common method bias. We performed analysis and checked where a single factor explains less than 50% variance in the data. The results revealed that a single factor explained 26.05% variance in the data, which means common method bias does not threaten the collected data’s credibility.

### 3.2. Assessment of Convergent Validity

The internal consistency of the data was measured to evaluate the quality of the collected data. We analyzed the internal consistency of the data using Cronbach’s alpha values. The values of Cronbach’s were above 0.70 for all the mentioned constructs, thus meeting the thresholds ≥0.70. The composite reliability (CR) is the best criterion for assessing the internal consistency of data [86]. Consequently, we also considered the internal consistency of the data by CR values, which show that all the values of the constructs exceed 0.70, endorsing the internal consistency of the data. We then measured the convergent validity, which signifies the degree to which one construct correlates with another [87]. Convergent validity is confirmed when the (AVE) of all the constructs is equal to or above 0.50, and the external loading is equal to or above 0.70 [87]. Established on the criteria, the AVE values for all the constructs are greater than 0.50, and the CR values are more significant than 0.70, endorsing the convergent validity of the data. The reliability analysis and convergent validity data are presented in Table 3.

### 3.3. Discriminant Validity

Discriminatory validity degree refers to that one construct is not related to another construct [5]. In this study, we used Fornell and Larcker’s (1981) and Heterotrait-Monotrait Ratio (HTMT) criterion to measure the discriminant validity [87,88]. According to Fornell and Larcker (1981), the criterion squares of AVEs should be greater than the values of the corresponding correlations. Table 4 shows the discriminant validity, which approves the existence of discriminant validity in our data as squares of AVEs that are greater than correlations among the constructs. To confirm the presence of discriminant validity, the constructs values must be less than 0.85 [88]. Table 5 shows that all constructs values are less than 0.85 thus confirming the discriminant validity as per HTMT criterion. 

### 3.4. Predictive Power of the Inner Model

The internal model fit criterion is assessed in two ways: the predictive compatibility of the model that is determined by the coefficient of determination (R2) and the value of cross-validated redundancy (Q2) [89]. The value of R2 represents the change in the endogenous structures that are explained by exogenous structures. In this study, the value of R2 for the endogenous construct is 47.9%, demonstrating moderate to high predictive accuracy. Next, we evaluated the value of cross-validated redundancy (Q2) by the blindfolding technique. The value of Q2 exceeding zero shows the predictive relevance in the model. The results show that the value of Q2 for the endogenous construct is 35.7%, which guarantees the predictive relevance to the study’s proposed model.

### 3.5. Hypotheses Testing

PLS-SEM has been applied to verify the hypothetical relationship through using the 2000 bootstrapping sampling method that was suggested by [89]. This study contains eleven hypotheses. The study results revealed that all the proposed hypotheses were supported. HI, H2, H3, and H4 posited positive influence of ATD (β = 0.174, t = 3.796, *p* < 0.05), SN (β = 0.327, t = 7.157, *p* < 0.05), PBC (β = 0.318, t = 6.863, *p* < 0.05), and MN (β = 0.183, t = 4.746, *p* < 0.05) on students’ intention to use reusable single cups were supported. The positive influence of TPB constructs signify the important role of TPB predicting the behavioral intention. H5, H6, and H7 posited positive influence of EC on ATD (β = 0.213, t = 3.553, *p* < 0.05), SN (β = 0.137, t = 2.132, *p* < 0.05), and MN (β = 0.265, t = 5.149, *p* < 0.05) were supported. H8, H9, H10, and H11 posited positive influence of GUI on ATD (β = 0.230, t = 4.132, *p* < 0.05), SN (β = 0.273, t = 5.370, *p* < 0.05), PBC (β = 0.280, t = 4.516, *p* < 0.05), and MN (β = 0.425, t = 8.860, *p* < 0.05) were also supported. Overall, the results indicate the effectiveness of the extended TPB model in predicting students’ intention to use reusable cups in higher education institutions. The detailed results of the hypotheses are presented in Table 6. 

## 4. Discussions

Promoting environmental sustainability is an emerging issue worldwide. Practitioners and environmentalists encourage people to adopt pro-environmental behavior and contribute to environmental sustainability. However, implementing environmental strategies is slow in developing countries, especially in Pakistan. This study aims to understand students’ intention to use reusable single cups in the university. The study results indicate that ATD, SN, and PBC are significant predictors of using single reusable cups in university campuses. These findings match the previous studies [5,34,41]. These results indicate that when people have the aptitude, tendency, and control to contribute to environmental quality, they are likely to use reusable single cups. The moral norm as an additional construct in the TPB model that significantly influences intention. The positive link between the moral norm and intention is in line with past researchers’ findings and offers conclusive evidence that the moral norm is a significant predictor of intention [27]. The findings also confirmed the positive influence of environmental concern on ATD, SN, and MN. These findings are consistent with previous researchers that emphasized the significant role of ATD, SN, and MN in predicting pro-environmental behaviors [5,26,29]. Previous studies suggest that Pakistani’s are philanthropic due to strict religious affiliation; therefore, they are inclined to contribute to improving environmental health [26]. Contrary to our expectations, the results indicate an insignificant impact of EC on PBC. 

Further, the results confirmed the positive influence of GUI on ATD, SN, PBC, and MN. The results match the findings of the previous researchers [69,75]. These findings indicate that promoting green initiatives at the institutional level fosters pro-environmental behavior. In addition, the results reveal that green initiatives that are adopted by Pakistani universities have increased the sense of responsibility and ability to contribute to the environment’s sustainability. This study provides a holistic understanding of using reusable cups to promote environmental health. This study’s findings established that personal and environmental factors shape people’s pro-environmental behavior.

## 5. Conclusions

The aim of the present study was to examine students’ intention to use reusable cups to promote environmental sustainability. The current study contributes to understanding the efforts of universities’ green initiatives on students’ pro-environmental behavior. The current study incorporates green university initiatives, environmental concern, and moral norms as the additional antecedents of TPB to explain students’ behavioral intention of using reusable cups. Past studies revealed that moral norms and environmental concern are essential elements of pro-environmental behavior [26,27,29]. In addition, some studies have examined the influence of universities/campuses green initiatives on environmental sustainability. However, prior studies lack a comprehensive model that examines GUIs influence on students’ intention to use reusable cups. This study fills the gap by integrating universities green initiatives in the extended TPB model to better understand students’ intention of using reusable cups. The data of the students has been collected from the five main cities of Pakistan where most of the large and medium size universities are located. A total of 361 useable responses were analyzed through PLS-SEM. The study results show that GUIs, PBC, and SN were the strongest factors that affect students’ intention to use reusable cups. In addition, the results revealed that moral norm is a significant factor that leads to using reusable cups for environmental sustainability. This study contributes theoretically by establishing a link between GUIs and TPB constructs and also confirmed the findings of Armitage and Conner’s meta-analysis regarding the inclusion of moral norm in the TPB model. Practically, this study acknowledged the role of GUIs as the center of environmental knowledge creation and the advocate of sustainability. 

## 6. Contributions & Implications

The current study has theoretical and practical implications. Theoretically, this study contributes to the environmental literature and theory. First, this study has established the relationship between ATD, SN, PBC, and MN on the intention to use reusable cups. Past studies have also established a link between TPB constructs, MN, and the intention to use environmental products [14,29,43]. Secondly, previous studies have comprehensively discussed the relationship between environmental concern, moral norms, and the core constructs of TPB in shaping pro-environmental behavior [29]. However, researchers have ignored the link between environmental concern and moral norms, particularly for reusable products. Therefore, this is the first study that confirmed the relationship between environmental concern and moral norms that are related to the use of reusable products. According to Armitage and Conner’s meta-analysis, the efficacy of TPB would be increased by the additional constructs, particularly the addition of moral norms in TPB would increase the efficacy of pro-environmental behavior [27]. This study confirms that adding moral norms has positively influenced the intention to use reusable cups. In addition, green university initiatives have a significant impact on the intention to use reusable cups. The efficacy of GUI in predicting pro-environmental behavior was also confirmed in the past studies [90]. This study integrated the GUI construct in TPB and provided evidence of the relationship between GUI and extended TPB constructs (ATD, SN, PBC, and MN). In addition, the study significantly contributes to the literature by highlighting the significant role of green universities initiatives on environmental behavior. The findings of the study revealed that the integration of green initiatives across various courses enhanced students green behavior. 

Practically, this study has multiple implications for practitioners and marketers. Despite advancements in environmentally sustainable practices worldwide, developing nations such as Pakistan are far behind in this regard. The wave of sustainability in developing nations is gradually increasing with the sense of responsibility, environmental concern, and the initiatives that are taken by the incumbent institutions [17,19]. In view of the findings of this study, several implications emerge to support the use of reusable cups. The study findings revealed that subjective norms are one of the most significant factors influencing the intention to use reusable cups. In Pakistan, subjective norms still play an essential role in many aspects of life, including personal preferences [26]. Educating society about the use of reusable cups and their positive impact on environmental health could help use them. In Pakistan, the manufacturer of reusable cups rarely advertises the products on media and other social platforms. Therefore, marketers should advertise reusable cups with a focus on the health of the environment and family to reinforce the adoption of reusable cups. The strong impact of PBC on the intention to adopt reusable cups indicates easy-to-own reusable cups. The Pakistani market is price sensitive, and the high prices of reusable cups could be a potential barrier to adopting reusable cups. The adoption of reusable cups can significantly increase by providing rewards and financial incentives for the use of reusable cups. In addition, the government should provide subsidies or tax reductions to the manufacturer for producing the reusable cups and selling them at lower prices to the public. These measures help to reduce the prices and increase the adoption of reusable cups. The study’s findings also revealed the strong influence of GUIs on the moral norm. GUIs are constantly working to highlight sustainability issues through seminars and workshops. Pakistan’s billion trees plantation campaign was successful due to the green initiatives of Pakistani universities that influenced the moral norm of the students. To further strengthen the moral norm, GUIs should highlight the impact of using single-useable cups on environmental pollution. To foster green initiatives in HEIs, universities must integrate and incorporate environmental issues across various courses that enhance students’ pro-environmental intentions. The learning programs should be based on a change process where students gradually learn the impact of green initiatives on environmental sustainability. Researchers recommended that students’ active participation and extensive research play a pivotal role. Therefore, investing in research regarding sustainable initiative increases students’ involvement in sustainable issues [90]. 

## 7. Limitations and Future Research

Although the current study has filled the gap in several dimensions, there are several limitations to this study. First, this study is quantitative, and data were collected from a self-administered questionnaire. For a comprehensive understanding of using reusable cups in a developing nation, in-depth interviews should be conducted to understand the factors affecting people’s intention to use reusable cups. Further, this study has used the TPB model that includes moral norms, environmental concerns, and green initiatives that may limit understanding of the pro-environmental tendency of people. Future studies can integrate TPB with the norm activation model to better understand people using reusable cups. The study’s findings are also limited as the data were collected through purposive sampling. Future studies can use probability data collection techniques to apply study findings broadly. Last but not least, Future studies can explore students’ participation in green initiatives and their impact on green behavior. In addition, researchers can explore the impact of green universities on students’ green entrepreneurial intention and sustainable product development because universities’ educational programs provide environmental knowledge and develop entrepreneurial skills.

## Figures and Tables

**Table 1:** Demographic profile.

		Frequency	Percentage
Gender	Male	142	30.3%
	Female	219	60.7%
Age	Under 25 years	47	13.01%
	26 to 35 years	156	43.2%
	36 to 45 years	148	41.0%
	Above 45 years	10	2.8%
Qualification	Intermediate	44	12.2%
	Bachelor	153	42.4%
	Master/MPhil	152	42.1%
	Ph.D.	12	3.3%
Household Income	0 PKR to 30,000 PKR	150	41.6%
	30,001 PKR to 60,000 PKR	62	17.2%
	60,001 PKR to 90,000 PKR	33	9.1%
	90,001 PKR to 12,000 PKR	39	10.8%
	120,001 PKR to 150,000 PKR	29	8%
	150,001 PKR or more	48	13.3%

**Table 2:** Constructs’ measurement.

Constructs	Items	Sources
Attitude	4	Waris et al. (2022) [34]
Subjective Norm	5	Hua and Wang (2019) [80]
Perceived Behavioral Control	5	Khan et al. (2019) [5]
Moral Norms	3	Soomro et al. (2022) [81]
Environmental Concern	6	Botetzagias et al. (2015) [82]
Green University Initiative	5	Al-Swidi and Saleh (2021) [83]; Ribeiro et al. (2021) [18]
Reusable Intention	5	Keller et al. (2021) [10]; Novoradovskaya et al. (2021) [7]

**Table 3:** Descriptive Analysis and Measurement Model.

Constructs	Items	Loading	Cronbach’s Alpha	CR	AVE
Attitude	ATD1	0.915	0.881	0.919	0.739
	ATD2	0.820			
	ATD3	0.898			
	ATD4	0.801			
Subjective Norm	SN1	0.768	0.912	0.935	0.742
	SN2	0.829			
	SN3	0.880			
	SN4	0.907			
	SN5	0.914			
Perceived Behavioral	PBC1	0.840	0.904	0.928	0.720
Control	PBC2	0.837			
	PBC3	0.846			
	PBC4	0.916			
	PBC5	0.906			
Moral Norm	MN1	0.880	0.788	0.875	0.701
	MN2	0.863			
	MN3	0.764			
Environmental Concern	EC1	0.743	0.836	0.877	0.543
	EC2	0.746			
	EC3	0.720			
	EC4	0.719			
	EC5	0.750			
	EC6	0.745			
Green University Initiatives	GUI1	0.746	0.854	0.902	0.698
	GUI2	0.881			
	GUI3	0.837			
	GUI4	0.871			
Reusable Intention	RI1	0.867	0.921	0.941	0.761
	RI2	0.823			
	RI3	0.846			
	RI4	0.916			
	RI5	0.906			

**Note:** ATD = Attitude, SN = Subjective norms, PBC = Perceived behavioral control, MN = Moral norms, EC = Environmental concern, GUI = Green university initiatives, RI = Reusable intention.

**Table 4:** Discriminant Validity (Fornell and Larcker criterion).

Latent Variables	1	2	3	4	5	6	7
Attitude	**0.860**						
Environmental Concern	0.281	**0.737**					
Green University Initiative	0.294	0.304	**0.835**				
Moral Norm	0.292	0.395	0.504	**0.837**			
Perceived Behavioral Control	0.129	0.112	0.280	0.231	**0.849**		
Reusable Intention	0.352	0.340	0.395	0.407	0.512	**0.872**	
Subjective Norm	0.255	0.220	0.314	0.303	0.394	0.552	**0.861**

**Note:** The diagonals (in bolds) represent the square root of AVE, and the off-diagonal values represent the correlations of each construct with other constructs. All correlations are statistically significant (*p* < 0.01).

**Table 5:** Discriminant Validity (Fornell and Larcker criterion).

Latent Variables	1	2	3	4	5	6	7
Attitude							
Environmental Concern	0.307						
Green University Initiative	0.336	0.333					
Moral Norm	0.340	0.450	0.612				
Perceived Behavioral Control	0.143	0.195	0.314	0.260			
Reusable Intention	0.387	0.362	0.444	0.459	0.556		
Subjective Norm	0.285	0.240	0.353	0.343	0.428	0.596	

**Table 6:** Hypotheses Assessment Summary.

Hypotheses	Beta	*p*-Values	t-Values	Decision	Effect
H1: ATD → RI	0.174	0.000	3.724	Supported	Small
H2: SN → RI	0.327	0.000	7.274	Supported	Large
H3: PBC → RI	0.318	0.000	6.755	Supported	Large
H4: MN → RI	0.183	0.000	4.528	Supported	Small
H5: EC → ATD	0.212	0.000	3.500	Supported	Small
H6: EC → SN	0.138	0.012	2.501	Supported	Small
H7: EC → MN	0.267	0.000	5.215	Supported	Large
H8: GUI → ATD	0.230	0.000	4.320	Supported	Small
H9: GUI → SN	0.272	0.000	5.245	Supported	Large
H10: GUI → PBC	0.271	0.000	4.427	Supported	Small
H11: GUI → MN	0.423	0.000	8.864	Supported	Large

**Note:** Path coefficients (Beta); significant at *p* < 0.05.

## Data Availability

The datasets analyzed during the current study are available from the corresponding author on reasonable request.
